# The MHD Newtonian hybrid nanofluid flow and mass transfer analysis due to super-linear stretching sheet embedded in porous medium

**DOI:** 10.1038/s41598-021-01902-2

**Published:** 2021-11-18

**Authors:** U. S. Mahabaleshwar, T. Anusha, M. Hatami

**Affiliations:** 1 Department of Mathematics, Davangere University, Shivagangothri, Davangere, 577 007 India; 2 Department of Mechanical Engineering, Ferdowsi University of Mashhad, Mashhad, Iran

**Keywords:** Applied mathematics, Computational science, Computational nanotechnology, Fluid dynamics

## Abstract

The steady magnetohydrodynamics (MHD) incompressible hybrid nanofluid flow and mass transfer due to porous stretching surface with quadratic velocity is investigated in the presence of mass transpiration and chemical reaction. The basic laminar boundary layer equations for momentum and mass transfer, which are non-linear partial differential equations, are converted into non-linear ordinary differential equations by means of similarity transformation. The mass equation in the presence of chemical reaction is a differential equation with variable coefficients, which is transformed to a confluent hypergeometric differential equation. The mass transfer is analyzed for two different boundary conditions of concentration field that are prescribed surface concentration (PSC) and prescribed mass flux (PMF). The asymptotic solution of concentration filed for large Schmidt number is analyzed using Wentzel-Kramer-Brillouin (WKB) method. The parameters influence the flow are suction/injection, superlinear stretching parameter, porosity, magnetic parameter, hybrid nanofluid terms, Brinkman ratio and the effect of these are analysed using graphs.

## Introduction

The behavior of the boundary layer due to continuous stretching sheet problem has a significant role in the industrial field and the cooling of porous sheet by polymer extrusion through stagnant liquid and in the characteristics of fluid motion over stretching sheet due to viscous flow is an important problem in the polymer industry. The analysis of concentration field with chemical reaction problem has got importance in most of the physical problems. The inclusion of the hybrid nanofluid for the fluid flow gives us the more efficient in increase of rate of heat transfer, that is rate of heating/cooling.

Vajravelu^1^ studied on the flow and heat transfer behavior due to impermeable stretching sheet embedded in saturated porous media with PST and PHF case of temperature and heat generation/absorption. Siddheshwar and Mahabaleshwar^2^ studied the flow and heat transfer due to nonlinear stretching sheet in PST and PHF case of wall temperature and asymptotic limit for small and large Prandtl number is studied using WKB approximation.

There are many works related to quadratically stretching sheet viz, Kumaran and Ramanaiah^3^ investigate on the viscous flow due to stretching sheet with quadratic velocity and with the linear mass flux of the sheet. Further they got the closed form solution with the effect of linear mass flux. Abel et al.^4^ evaluated the solution for heat transfer of the viscoelastic fluid flow due to isothermal stretching surface considering the magnetic field effect and heat generation. Further they obtain the asymptotic limits for small and large value of Prandtl number and the work revealed that in the case of small Prandtl number the viscoelasticity impact and the magnetic field will decrease the temperature field. Further Kelly et al.^5^ also studied the heat and mass transfer asymptotic limit for small and large Schmidt number. Similarly Kelson^6^ studied viscous flow with quadratic stretching sheet and Kumaran et al.^7^ also studied the same with linearly permeable surface, magnetic field effects and mass transpiration and obtained that the phenomena of shear thinning will reduced the shear stress of wall.

Turkyilmazoglu^8^ works on the consequences of Dufour and Soret on the MHD flow and the heat transfer of viscoelastic fluid through the vertical stretching surface embedded in the porous medium and found the unique/multiple solution and existence/nonexistence of solution by the influence of considering parameters. Recently the same effect for mixed convective flow with radiation is studied by Mahabaleshwar et al.^9^ and Patil^10^ studied the couple stress fluid flow for first order chemical reaction. Aly et al.^11^ also examine the boundary layer MHD flow due to stretching surface embedded in porous medium with the effect of second order slip using ChPDM technique results that magnetic field, porosity, slip parameters reduces the thickness of nano boundary layer. Wu^12^ study the boundary layer gas flow over linearly stretching/shrinking sheet and theoretically prove that the induced velocity slip by the effect of mass transfer will significantly change the velocity of the gas flow, further there is considerable variation in the temperature field and heat flux because of the convection phenomena.

Nagaraju et al.^13^ use the ADM and Pade approximation method to get the solution to the nonlinear differential equation with unsteady boundary layer flow and porous media. Turkyilmazoglu^14^ made the mathematical approach by deriving the formulas to show how Buongiorno nanofluid model will reduce/enhance the heat and mass transfer and are well agree with the previous works. Many works done on the mass transfer with chemical reaction such as, Andersson et al.^15^ studied it over stretching sheet and obtain that the thickness of concentration boundary layer will reduce and mass transfer rate will enhance with the destructive chemical reaction. Further the similar problem solved by Siddheshwar et al.^16^ for analytical solution due to stretching sheet problem. Andersson and Valnes^17^ examined the flow and heat transfer of ferrofluid due to stretching sheet in consideration of magnetic dipole results in that the fluid motion will decelerate and heat transfer rate will reduces by the effect of magnetic field.

Mahabaleshwar et al.^18^ studied the impact of radiation and mass transpiration on the MHD unsteady flow and heat transfer due to linear stretching sheet by applying two kinds of boundary conditions for temperature i.e., PTDCST and PTDWHF and obtain dual solution in both stretching and shrinking boundary. Cortell^19^ studied it with steady MHD over permeable stretching sheet with quadratic velocity. Further Andersson^20^ got the exact analytical solution for the momentum conservation problem which is valid for all values of Reynolds number. The flow and heat transfer over shrinking sheet also considered as significant problem in the industrial field. Fan and Zhong^21^ studied the boundary layer flow and heat transfer due to shrinking surface concerned by the arbitrary velocity distribution. In 1986 Siddappa and Abel^22^ investigates the flow of Walters’ liquid B over stretching sheet considering the effect of suction and Nayakar et al.^23^ investigates on the same work for nonlinear stretching/shrinking sheet and with MHD. Mahabaleshwar et al.^24^ also studied on the same for MHD flow with first order slip and mass transfer and also some researchers studied different physical parameters^25–35^.

In 1992 Vajravelu and Rollins^36,37^ analyze the flow and heat transfer of electrically conducting fluid due to stretching sheet and flow of second order fluid respectively, with PST and PHF cases of wall temperature and obtained asymptotic limits for large Prandtl number. Further Vajravelu and Cannon^38^ studied it due to the porous medium and establish the existence and uniqueness of the solution. Mahabaleshwar et al.^39^ made the contribution on the inclined MHD flow, mass transfer and heat transfer with radiation effect. Mahabaleshwar et al.^40^ made the article on the MHD flow with carbon nanotubes and effect of mass transpiration and radiation on it. Anusha et al.^41^ investigates the unsteady inclined MHD flow for Casson fluid with hybrid nanoparticles.

In this paper, we consider the effect of MHD and concentration with first order chemical reaction, mass transpiration of incompressible hybrid nanofluid flow due to porous stretching surface with quadratic velocity. The mass transfer is analyzed for two different boundary conditions of concentration field that are prescribed surface concentration (PSC) and prescribed mass flux (PMF). The asymptotic solution of concentration filed for large Schmidt number is analyzed using Wentzel–Kramer–Brillouin (WKB) method.

## Mathematical formulation

**Figure 1:**
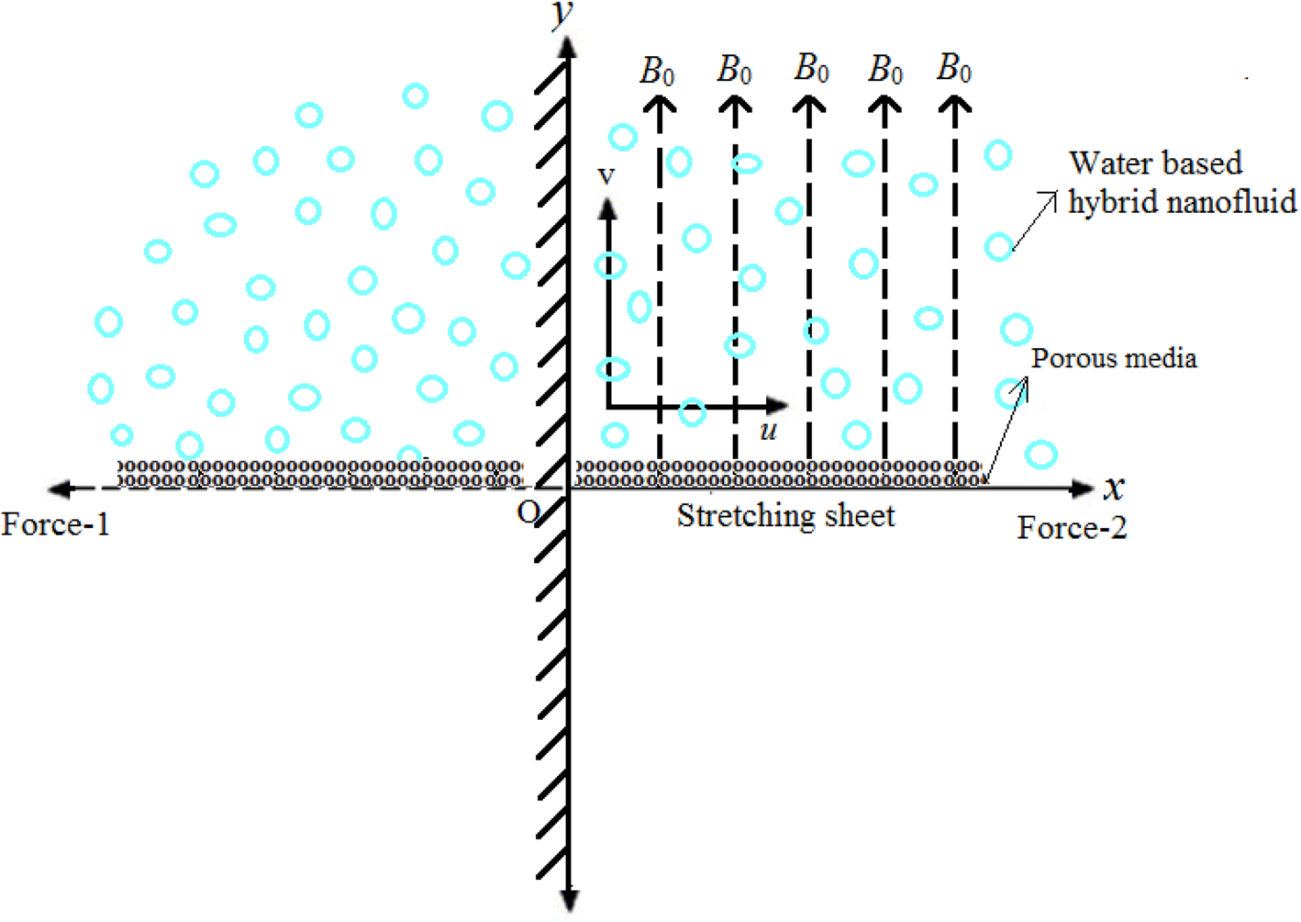
Schematic diagram representing the flow problem.

Consider the steady 2-D incompressible hybrid nanofluid flow due to the stretching sheet with quadratic velocity embedded in the porous media with effective viscosity as shown in the Fig. 1. The flow is along *x*- direction and *y*- direction is perpendicular to it along which the magnetic field with strength *B*_0_ is applied. The velocity of stretching/shrinking sheet is proportional to the square of the distance of a point from the origin *O*.

### Momentum problem

The governing continuity and Navier- Stoke equations for the present flow are given as (see Kumaran and Ramanaiah 1996, Neil 2011. Siddheshwar and Mahabaleshwar 2018),

1 $$ \frac{\partial u}{{\partial x}} + \frac{\partial v}{{\partial y}} = 0, $$

2 $$ u\frac{\partial u}{{\partial x}} + v\frac{\partial u}{{\partial y}} = \nu_{eff} \frac{{\partial^{2} u}}{{\partial y^{2} }} - \frac{{\sigma_{hnf} B_{0}^{2} }}{{\rho_{hnf} }}u - \frac{{\mu_{hnf} }}{{\rho_{hnf} K}}u, $$

subject to the boundary conditions,

3a $$ u = ax + bx^{2} \,\,,\,\,v = v_{c} + \Delta \,x,\quad {\text{at}}\;y = 0, $$

3b $$ u \to 0\,\,,\,\,{\text{as}}\,\,y \to \infty , $$

with defined stream function as,

4 $$ \psi = \sqrt {a\nu_{f} } xf\left( \eta \right) - \frac{c}{2}x^{2} f_{\eta } \left( \eta \right),\,\,{\text{where}}\,\,\eta = \sqrt {\frac{a}{{\nu_{f} }}} y $$

here $a,b,\Delta$ are constants. Equation (3b) implies that as $y \to \infty$ the liquid has no lateral motion.

5a $$ U = \frac{u}{{\sqrt {a\nu_{f} } }}\,\,,\,\,V = \frac{{\text{v}}}{{\sqrt {a\nu_{f} } }}\,\,,\,\,X = x\sqrt {\frac{a}{{\nu_{f} }}} \,\,,\,\,Y = y\sqrt {\frac{a}{{\nu_{f} }}} , $$

5b $$ b^{*} = \frac{b}{a}\sqrt {\frac{{\nu_{f} }}{a}} \,\,,\,\,\Delta^{*} = \frac{\Delta }{2a}\,\,,\,\,{\text{V}}_{C}^{*} = \frac{{{\text{v}}_{c} }}{{\sqrt {a\nu_{f} } }}\,\,,\,\,\psi^{*} = \frac{\psi }{{\nu_{f} }}, $$

In Eqs. (1)-(3), the transformed governing equations are obtained as follows:

6 $$ \frac{\partial U}{{\partial X}} + \frac{\partial V}{{\partial Y}} = 0, $$

7 $$ U\frac{\partial U}{{\partial X}} + V\frac{\partial U}{{\partial Y}} = \Lambda \frac{{\partial^{2} U}}{{\partial Y^{2} }} - \frac{{D_{3} }}{{D_{1} }}MU - \frac{{D_{2} }}{{D_{1} }}Da^{ - 1} U, $$

where $M = \frac{{\sigma_{hnf} B_{0}^{2} }}{{a\rho_{hnf} }}$, $Da^{ - 1} = \frac{{\nu_{f} }}{aK}$ are magnetic parameter and inverse Darcy number. The B.Cs (3a and 3b) become,

8a $$ U = X + b^{*} {\kern 1pt} X^{2} \,\,,\,\,V = {\text{V}}_{C}^{*} + 2\Delta^{*} X,\quad {\text{at}}\;Y = 0, $$

8b $$ U \to 0,\quad {\text{as}}\;Y \to \infty $$

using Eqs. (4), (5),

9a $$ \psi^{*} = Xf\left( Y \right) - \Delta^{*} {\kern 1pt} X^{2} f_{Y} \left( Y \right),\,\,X = \xi = x\sqrt {\frac{a}{{\nu_{f} }}} \,\,\,,\,\,Y = \eta = \sqrt {\frac{a}{{\nu_{f} }}} y, $$

9b $$ U = Xf_{Y} - \Delta^{*} {\kern 1pt} X^{2} f_{YY} ,\,\,V = - f + 2\Delta^{*} Xf_{Y} , $$

where subscript *Y* denotes the derivative w.r.to *Y*.

Substitution of Eqs. (8), (9) in Eq. (7) and results in the following nonlinear ordinary differential equations:

10a $$ \Lambda f_{YYY} - f_{Y}^{2} + ff_{YY} - \frac{1}{{D_{1} }}\left( {D_{3} M + D_{2} Da^{ - 1} } \right)f_{Y} = 0, $$

10b $$ \Lambda f_{YYYY} - f_{Y} f_{YY} + ff_{YYY} - \frac{1}{{D_{1} }}\left( {D_{3} M + D_{2} Da^{ - 1} } \right)f_{YY} = 0, $$

and

10c $$ f_{Y} f_{YYY} - f_{YY}^{2} = 0. $$

Here

$$ D_{1} = \frac{{\rho_{hnf} }}{{\rho_{f} }} = \left( {1 - \varphi_{2} } \right)\left( {1 - \varphi_{1} + \varphi_{1} \frac{{\rho_{{s_{1} }} }}{{\rho_{f} }}} \right) + \varphi_{2} \left( {\frac{{\rho_{{s_{2} }} }}{{\rho_{f} }}} \right), $$

$$ D_{2} = \frac{{\mu_{hnf} }}{{\mu_{f} }} = \frac{1}{{\left( {1 - \varphi_{1} } \right)^{2.5} \left( {1 - \varphi_{2} } \right)^{2.5} }}, $$

10d $$ D_{3} = \frac{{\sigma_{hnf} }}{{\sigma_{f} }} = \frac{{\sigma_{{s_{2} }} + 2\sigma_{bf} + 2\varphi_{2} \left( {\sigma_{{s_{2} }} - \sigma_{f} } \right)}}{{\sigma_{{s_{2} }} + 2\sigma_{bf} - \varphi_{2} \left( {\sigma_{{s_{2} }} - \sigma_{f} } \right)}},\;{\text{where}}\;\sigma_{bf} = \sigma_{f} \frac{{\sigma_{{s_{1} }} + 2\sigma_{f} + 2\varphi_{1} \left( {\sigma_{{s_{1} }} - \sigma_{f} } \right)}}{{\sigma_{{s_{1} }} + 2\sigma_{f} - \varphi_{1} \left( {\sigma_{{s_{1} }} - \sigma_{f} } \right)}}. $$

Equation (11) can also obtained by differentiating Eq. (10) with respect to *Y* and from the B.Cs (8a & b) satisfied by *f* can be obtained as,

11 $$ f\left( 0 \right) = - {\text{V}}_{C}^{*} ,\quad f_{Y} \left( 0 \right) = 1,\quad f_{YY} \left( 0 \right) = - \frac{{b^{*} }}{{\Delta^{*} }},\quad f_{Y} \left( \infty \right) = 0,\quad f_{YY} \left( \infty \right) = 0. $$

The solution of Eq. (10a) is in the form as below,

12a $$ f\left( Y \right) = A + Be^{ - \alpha Y} , $$

where

12b $$ A = - {\text{V}}_{C}^{*} + \frac{1}{\alpha }\,\,{,}\,\,B = - \frac{1}{\alpha }\,\,{\text{and}}\,\,\alpha = \frac{{b^{*} }}{{\Delta^{*} }}, $$

On using these in Eq. (10a) gives,

13 $$ \Lambda \alpha^{2} + {\text{V}}_{C}^{*} \alpha - \frac{1}{{D_{1} }}\left( {D_{3} M + D_{2} Da^{ - 1} } \right) - 1 = 0, $$

It gives the relations,

14a $$ {\text{V}}_{C}^{*} = - \Lambda \alpha + \frac{1}{{\alpha D_{1} }}\left( {D_{3} M + D_{2} Da^{ - 1} } \right) + \frac{1}{\alpha }, $$

and

14b $$ \alpha = - \frac{{{\text{V}}_{C}^{*} }}{2\Lambda } \pm \frac{1}{2\Lambda }\sqrt {\left( {{\text{V}}_{C}^{*} } \right)^{2} + 4\Lambda + \frac{4\Lambda }{{D_{1} }}\left( {D_{3} M + D_{2} Da^{ - 1} } \right)} . $$

The flow pattern for $\psi^{*} = C$ gives the,

15 $$ Y = \frac{1}{\alpha }\log \left[ {\frac{{X\left( {\frac{1}{\alpha } + \Delta^{*} X} \right)}}{{\frac{X}{\alpha } - X\,V_{C}^{*} - C}}} \right]. $$

### Mass transfer analysis

16 $$ u\frac{\partial C}{{\partial x}} + {\text{v}}\frac{\partial C}{{\partial y}} = D_{B} \frac{{\partial^{2} C}}{{\partial y^{2} }} + k_{C} \left( {C - C_{\infty } } \right), $$

On using dimensionless transformation (5).

Here, *C* is the concentration field, *D*_*B*_ is molecular diffusivity, *K*_*C*_ is chemical reaction parameter and $C_{\infty }$ is the ambient concentration. Define, $C = C_{\infty } + \left( {C_{w} - C_{\infty } } \right)\phi \left( \eta \right)$.

By using the (5) and Eq. (16) will become,

17 $$ U\frac{\partial \phi }{{\partial X}} + {\text{V}}\frac{\partial \phi }{{\partial Y}} = \frac{1}{Sc}\frac{{\partial^{2} \phi }}{{\partial Y^{2} }} + \vartheta \,\phi = 0, $$

Here $Sc = \frac{{\nu_{f} }}{{D_{B} }}$ is Schmidt number and $\vartheta = \frac{{k_{C} }}{a}$ is chemical reaction parameter.

#### Prescribed surface concentration (PSC)

For PST, the defined B.Cs are,

18 $$ \phi \left( 0 \right) = 1\,\,{\text{and}}\,\,\phi \left( \infty \right) = 0, $$

Use the function transformation as, $\phi \left( \eta \right) = \beta X^{r} \Phi \left( Y \right)$, Eq. (17) will become,

19 $$ \frac{1}{Sc}\Phi_{YY} + \left( {f - 2\Delta^{*} Xf_{Y} } \right)\Phi_{Y} + \left( {\vartheta + \Delta^{*} Xf_{YY} - f_{Y} } \right)\Phi = 0, $$

On equating coefficients of $X^{0} \,{\text{and}}\,X$ in Eq. (19) gives the relations,

20a $$ \Phi_{YY} + Scf\Phi_{Y} - Sc\left( {rf_{Y} - \vartheta } \right)\Phi = 0, $$

20b $$ f_{YY} \Phi - \frac{2}{r}f_{Y} \Phi_{Y} = 0. $$

By Eq. (20b) we get,

21 $$ \Phi \left( Y \right) = \exp \left[ { - \alpha r\frac{Y}{2}} \right], $$

Using (12a and 12b) in (20a)

22 $$ \Phi_{YY} + Sc\left( {A + Be^{ - \alpha Y} } \right)\Phi_{Y} - Sc\left( {r\,e^{ - \alpha Y} - \vartheta } \right)\Phi = 0, $$

On using the transformation $\varepsilon = - \frac{Sc}{{\alpha^{2} }}e^{ - \alpha Y}$ Eq. (22) will transform to the form,

23 $$ \varepsilon \,\Phi_{\varepsilon \varepsilon } + \left( {1 - \frac{ASc}{\alpha } - \varepsilon } \right)\Phi_{\varepsilon } + Sc\left( {r + \frac{Sc\vartheta }{{\alpha^{2} \varepsilon }}} \right)\Phi = 0, $$

With corresponding transformed B.Cs as,

24 $$ \Phi \left( { - \frac{Sc}{{\alpha^{2} }}} \right) = 1\,\,{\text{and}}\,\,\Phi \left( 0 \right) = 0, $$

Then the solution of Eq. (23) in terms of $\varepsilon$ is,

25 $$ \Phi \left( \varepsilon \right) = \left( { - \frac{{\alpha^{2} }}{Sc}\varepsilon } \right)^{{\frac{{\chi_{1} + \chi_{2} }}{2}}} \frac{{F\left[ {\frac{{\chi_{1} + \chi_{2} }}{2} - r,1 + \chi_{2} ,\varepsilon } \right]}}{{F\left[ {\frac{{\chi_{1} + \chi_{2} }}{2} - r,1 + \chi_{2} , - \frac{Sc}{{\alpha^{2} }}} \right]}}, $$

And in terms of *Y* it will become,

26 $$ \Phi \left( Y \right) = exp\left[ { - \alpha Y\left( {\frac{{\chi_{1} + \chi_{2} }}{2}} \right)} \right]\frac{{F\left[ {\frac{{\chi_{1} + \chi_{2} }}{2} - r,1 + \chi_{2} , - \frac{Sc}{{\alpha^{2} }}e^{ - \alpha Y} } \right]}}{{F\left[ {\frac{{\chi_{1} + \chi_{2} }}{2} - r,1 + \chi_{2} , - \frac{Sc}{{\alpha^{2} }}} \right]}}, $$

Here $\chi_{1} = \frac{Sc\,A}{\alpha }\,\,,\,\,\chi_{2} = \frac{1}{\alpha }\sqrt {Sc^{2} A^{2} - 4Sc\vartheta }$ and $M\left[ {a,b;z} \right]$ denotes the confluent hyper geometric polynomial and Sherwood number, the dimensionless mass transfer rate is,

27 $$ - \Phi_{Y} \left( 0 \right) = \alpha \left( {\frac{{\chi_{1} + \chi_{2} }}{2}} \right) - \frac{Sc}{\alpha }\left( {\frac{{\frac{{\chi_{1} + \chi_{2} }}{2} - r}}{{1 + \chi_{2} }}} \right)\frac{{F\left[ {\frac{{\chi_{1} + \chi_{2} }}{2} - r + 1,2 + \chi_{2} , - \frac{Sc}{{\alpha^{2} }}} \right]}}{{F\left[ {\frac{{\chi_{1} + \chi_{2} }}{2} - r,1 + \chi_{2} , - \frac{Sc}{{\alpha^{2} }}} \right]}}, $$

The local mass flux can be expressed as,

28 $$ Q_{w} = - X^{r} \phi_{Y} \left( 0 \right), $$

#### Prescribed surface mass flux (PMF)

The corresponding B.Cs for PMF case are,

29 $$ \phi \left( 0 \right) = - 1\,\,{\text{and}}\,\,\phi \left( \infty \right) = 0, $$

Use the function transformation as, $\phi \left( \eta \right) = \beta X^{r} G\left( Y \right)$, Eq. (17) will become,

30 $$ \frac{1}{Sc}G_{YY} + \left( {f - 2\Delta^{*} Xf_{Y} } \right)G_{Y} + \left( {\vartheta + \Delta^{*} Xf_{YY} - f_{Y} } \right)G = 0, $$

On equating coefficients of $X^{0} \,{\text{and}}\,X$ in Eq. (30) gives the relations,

31a $$ G_{YY} + Sc\,f\,G_{Y} - Sc\left( {r\,f_{Y} - \vartheta } \right)G = 0, $$

31b $$ f_{YY} G - \frac{2}{r}f_{Y} G_{Y} = 0. $$

By Eq. (31b) we get,

32 $$ G\left( Y \right) = \left( {\frac{2}{\alpha r}} \right)\exp \left[ { - \alpha r\frac{Y}{2}} \right], $$

The solution of Eq. (31a) will become,

31 $$ G\left( Y \right) = \frac{{\left( {e^{ - \alpha Y} } \right)^{{m_{1} }} F\left[ {m_{1} - r,1 + \chi_{2} , - \frac{Sc}{{\alpha^{2} }}e^{ - \alpha Y} } \right]}}{{\alpha m_{1} F\left[ {m_{1} - r,1 + \chi_{2} , - \frac{Sc}{{\alpha^{2} }}} \right] - \frac{Sc}{\alpha }\left( {\frac{{m_{1} - r}}{{1 + \chi_{2} }}} \right)F\left[ {m_{1} - r + 1,2 + \chi_{2} , - \frac{Sc}{{\alpha^{2} }}} \right]}}, $$

where $$m_{1} = \frac{{\chi_{1} + \chi_{2} }}{2}$$.

## Wentzel-Kramer-Brillouin (WKB) method of asymptotic solution

### Asymptotic solution for large Schmidt number

WKB approximation is used to find out the matched asymptotic expansion (MAE) (as in Ref^38^) in the case of large Schmidt number for both PSC and PMF cases. And this is not possible to find MAE in case of small Schmidt number. In this case we can find analytic solution in PSC and PMF cases.

#### PSC

In PSC case the boundary layer equation with B.C is as follows,

32a $$ \Phi_{YY} + Scf\Phi_{Y} - Sc\left( {rf_{Y} - \vartheta } \right)\Phi = 0, $$

32b $$ \Phi \left( 0 \right) = 1\,\,{\text{and}}\,\,\Phi \left( \infty \right) = 0, $$

Take the substitution $Sc^{ - 1} = \Xi$ which is very small because of large Schmidt number, Eq. (32a) becomes,

33 $$ \Xi \Phi_{YY} + f\Phi_{Y} - \left( {rf_{Y} - \vartheta } \right)\Phi = 0, $$

Here $\Xi$ present in the highest order derivative which indicates the boundary layer behavior at $Y = 0$, from Eq. (12a)

34 $$ f\left( Y \right) = A + Be^{ - \alpha Y} , $$

here $$A = - {\text{V}}_{C}^{*} + \frac{1}{\alpha }\,\,{\text{and}}\,\,B = - \frac{1}{\alpha }\,\,$$, where $$A > 0\,{\text{and}}\,B < 0$$.

If $A > \left| B \right|$ then $f\left( Y \right) \ne 0$ for any $Y \in [0,\infty )$. But when $A < \left| B \right|$ then $f\left( Y \right) = 0$ for

35 $$ Y = Y^{*} = \frac{1}{\alpha }Ln\left[ {\left| \frac{B}{A} \right|} \right] $$

The behavior of solution of Eq. (33) is changes because of the point $Y = Y^{*}$. For the case $A = \left| B \right|$ the point $Y^{*} = 0$. By using WKB method, the uniform expansion is found for the cases $A > \left| B \right|\,{\text{and}}\,A < \left| B \right|$.

36 $$ {\text{Let}}\;\Phi \left( Y \right) = \exp \left[ { - \frac{1}{2\Xi }\int\limits_{0}^{Y} {f\left( z \right)dz} } \right]\Theta \left( Y \right), $$

Using (36) in (33) we get the most useful form,

37 $$ \Xi^{2} \Theta_{YY} - \left[ {\frac{1}{4}f^{2} + \Xi \left( {r + \frac{1}{2}} \right)f_{Y} - \vartheta \,\Xi } \right]\Theta = 0, $$

For small $\Xi$, a small uniform approximation as the limit in $\Xi$ is obtained by assuming the solution of Eq. (37) in the form,

38 $$ \Theta \left( Y \right) = \exp \left[ {\frac{1}{\Xi }\sum\limits_{{}}^{{}} {\Xi^{n} Q_{n} \left( Y \right)} } \right], $$

Using assumed solution (38) in (37) gives the relation,

39 $$ \left( {Q_{0}^{^{\prime}} } \right)^{2} + 2\Xi Q_{0}^{^{\prime}} Q_{1}^{^{\prime}} + \Xi Q_{0}^{^{\prime\prime}} - \vartheta \,\Xi - \frac{1}{4}f^{2} - \Xi \left( {r + \frac{1}{2}} \right)f^{^{\prime}} - O\left( {\Xi^{2} } \right) = 0, $$

In the above relation the terms with $O\left( 1 \right)\,{\text{and}}\,O\left( \Xi \right)$ are,

40a $$ \left( {Q_{0}^{\prime } } \right)^{2} = \frac{1}{4}f^{2} , $$

40b $$ 2Q_{0}^{\prime } Q_{1}^{\prime } + Q_{0}^{\prime \prime } - \vartheta \, - \left( {r + \frac{1}{2}} \right)f^{\prime } = 0, $$

The solution of Eq. (40a) is,

41 $$ Q_{0} \left( Y \right) = - \frac{1}{2}\int\limits_{0}^{Y} {f\left( z \right)dz} , $$

For the particular case $\vartheta = 0$, the relation obtained from eqs. (40b) and (41),

42 $$ Q_{1} = - \left( {r + 1} \right)Ln\left[ {f\left( z \right)} \right], $$

Use eqs. (41) and (42) in Eq. (38) gives,

43 $$ \Theta \left( Y \right) = C_{1} \left[ {f\left( z \right)} \right]^{{ - \left( {r + 1} \right)}} \exp \left[ { - \frac{1}{2\Xi }\int\limits_{0}^{Y} {f\left( z \right)dz} } \right], $$

Therefore from (36), the solution for $\Phi \left( Y \right)$ is,

44 $$ \Phi \left( Y \right) = C_{1} \left[ {f\left( z \right)} \right]^{{ - \left( {r + 1} \right)}} \exp \left[ { - \frac{1}{\Xi }\int\limits_{0}^{Y} {f\left( z \right)dz} } \right], $$

Using eqs. (32) and (34) in (44) will give the solution as,

45 $$ \Phi \left( Y \right) = \left( {\frac{A + B}{{A + Be^{ - \alpha Y} }}} \right)^{{\left( {r + 1} \right)}} \exp \left\{ { - \frac{1}{\Xi }\left[ {AY + \frac{B}{\alpha }\left( {1 - e^{ - \alpha Y} } \right)} \right]} \right\} + O\left( \Xi \right). $$

#### PMF

In PMF case the boundary layer equation with B.C is as follows,

46a $$ G_{YY} + Scf\,G_{Y} - Sc\left( {rf_{Y} - \vartheta } \right)G = 0, $$

46b $$ G_{Y} \left( 0 \right) = - 1\,\,{\text{and}}\,\,G\left( \infty \right) = 0, $$

The solution in this case becomes,

47 $$ G\left( Y \right) = \frac{{\Xi \left( {A + B} \right)^{{\left( {r + 2} \right)}} \left( {A + Be^{ - \alpha Y} } \right)^{{ - \left( {r + 1} \right)}} }}{{\left( {A + B} \right)^{2} - \Xi \,B\alpha \left( {r + 1} \right)}}\exp \left\{ { - \frac{1}{\Xi }\left[ {AY + \frac{B}{\alpha }\left( {1 - e^{ - \alpha Y} } \right)} \right]} \right\} + O\left( \Xi \right), $$

where $\Xi = \frac{1}{Sc}$ is small number, $A = - {\text{V}}_{C}^{*} + \frac{1}{\alpha }\,\,{\text{and}}\,\,B = - \frac{1}{\alpha }$.

## Results and discussion

The hybrid nanofluid flow through the superlinear stretching sheet embedded in porous media in the presence of MHD and the chemical reaction effect on the concentration field is investigated in the present flow. Impact of suction/ injection and the concentration distribution is studied in the case of PSC and PMF. And the concentration distribution for the large Schmidt number is analysed for both PSC and PMF case using WKB approximations. The stretching sheet can be realized in practice only with great care and meticulous effort, super-linear stretching sheet is a more practical problem. As a consequence exciting of the fluid as we go downstream along the sheet is to be expected. This is brought out quite explicitly in the current issue. The liquid is basically meant to cool the stretching sheet whose property as a final product depends greatly on the rate at which it is cooled. The problem is a prototype for many other practical problems also, akin to the polymer extrusion process, like.

- Drawing, annealing and tinning of copper wires,
- Continuous stretching, rolling and manufacturing of plastic film and artificial fibers,
- Extrusion of a material and heat-treated materials that travel between feed and wind-up rollers or on conveyor belts.

The delicate nature of the problem dictates the fact that the magnitude of the stretching rate has to be small. This also ensures that the stretching material released between the two solid blocks into the liquid continues to be a plane surface rather than a curved one. Mathematical manageability is therefore at its best in the problem.

**Figure 2:**
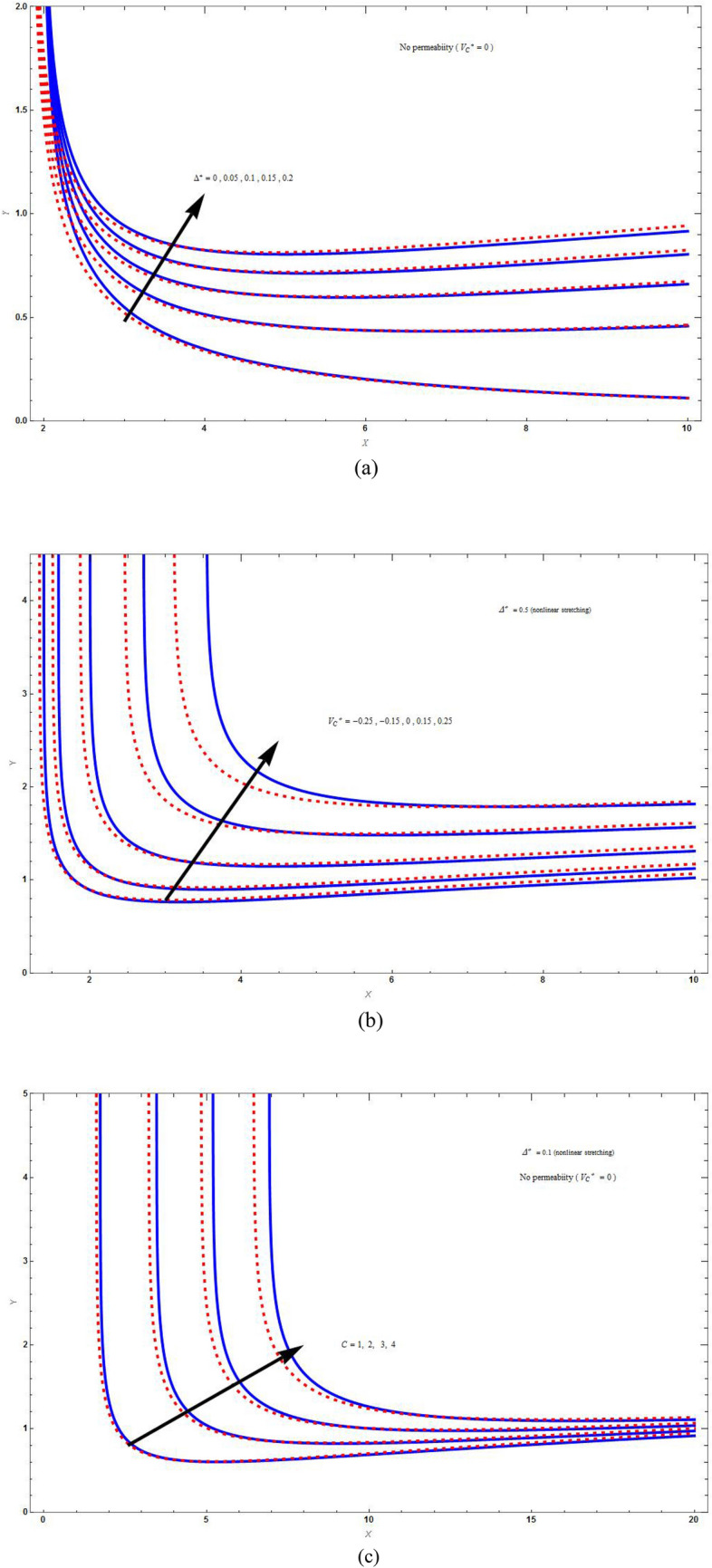
The streamline plot $\psi \left( {X,Y} \right) = C$ for varying (**a**) superlinear parameter $\left( {\Delta^{*} } \right)$ with $M = C = \Lambda = 1\,,\,K = 2$, (**b**) suction/injection parameter $\left( {V_{C}^{*} } \right)$ with $M = C = \Lambda = 1\,,\,K = 2$ and (**c**) values of *C* with $M = \Lambda = K = 1\,$. Blue solid lines are for the base fluid and red dotted lines denotes the hybrid nanofluid $Cu - Al_{2} O_{3} /{\text{water}}$.

**Figure 3:**
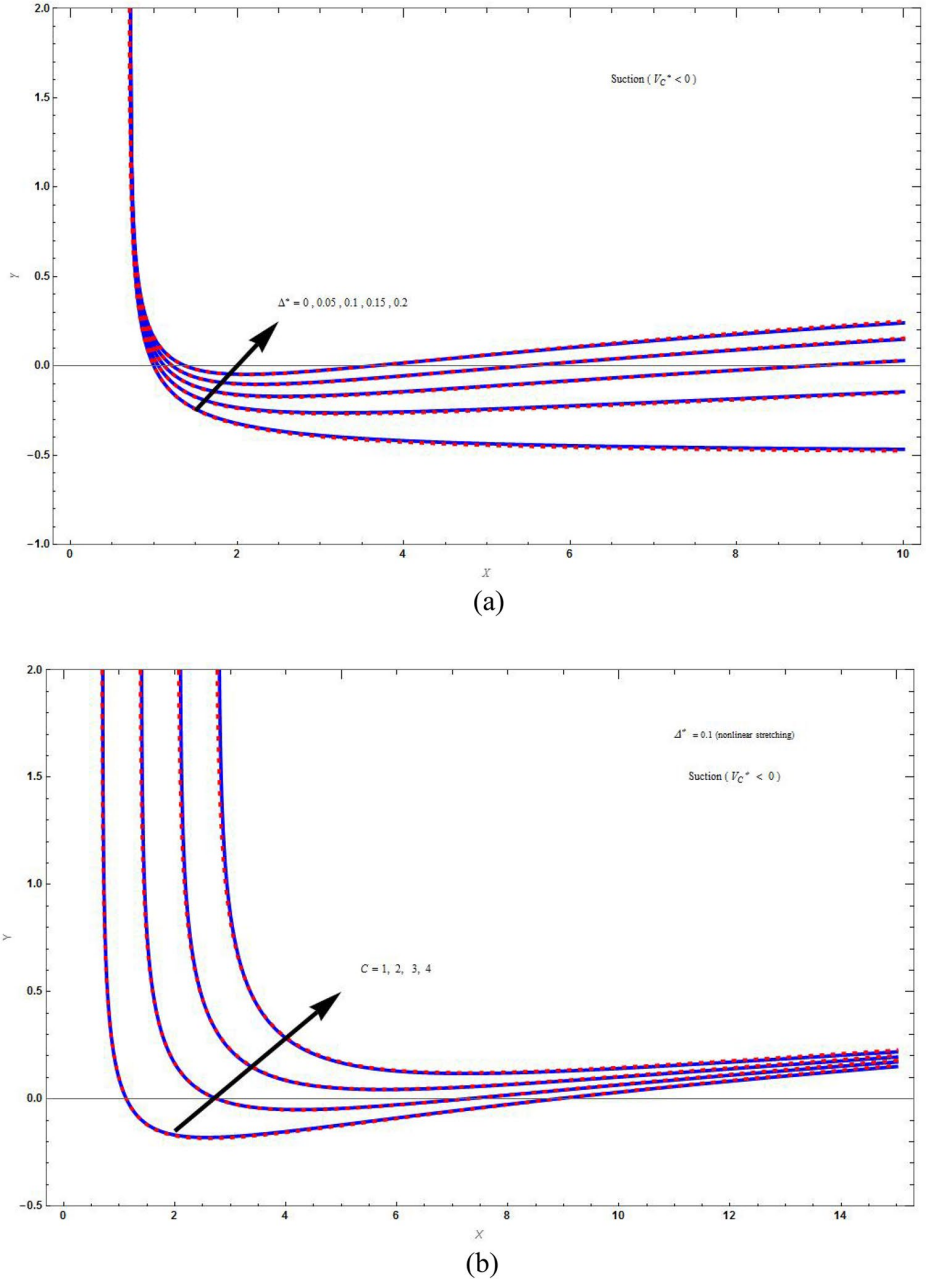
The streamline plot $\psi \left( {X,Y} \right) = C$ for suction boundary by varying (**a**) superlinear parameter $\left( {\Delta^{*} } \right)$ with $M = C = \Lambda = 1\,,\,K = 2$, and (**b**) values of *C* with $M = \Lambda = K = 1\,$. Blue solid lines are for the base fluid and red dotted lines denotes the hybrid nanofluid $Cu - Al_{2} O_{3} /{\text{water}}$.

Figure 2a and b are the plots for the streamline $\psi \left( {X,Y} \right) = 1$ for stretching sheet by varying respectively the values of superlinear parameter $\Delta^{*}$ and suction/injection parameter $V_{C}^{*}$. Figure 2a is plot for impermeable case and Fig. 3a is for suction case, which revealed that as the value of $\Delta^{*}$ increase, i.e., increasing in the rate of super-linear stretching results in enhancement of the liquid lift along downstream. The effect of increasing values of *C* on the streamline $\psi \left( {X,Y} \right) = C$ for the nonlinear stretching case is shown in Fig. 2c for impermeable boundary and Fig. 3b for suction boundary. Streamlines lifted up because of taking nonlinear stretching and are converging at the long distance along downstream. The liquid lift for base fluid is higher than that of hybrid nanofluid. These plots demonstrate that extent of liquid along the vertical sheet increases with increase in axial distance. This is because of the reason that the reaction of the liquid does not match to the stretching when there is nonlinear stretching.

**Figure 4:**
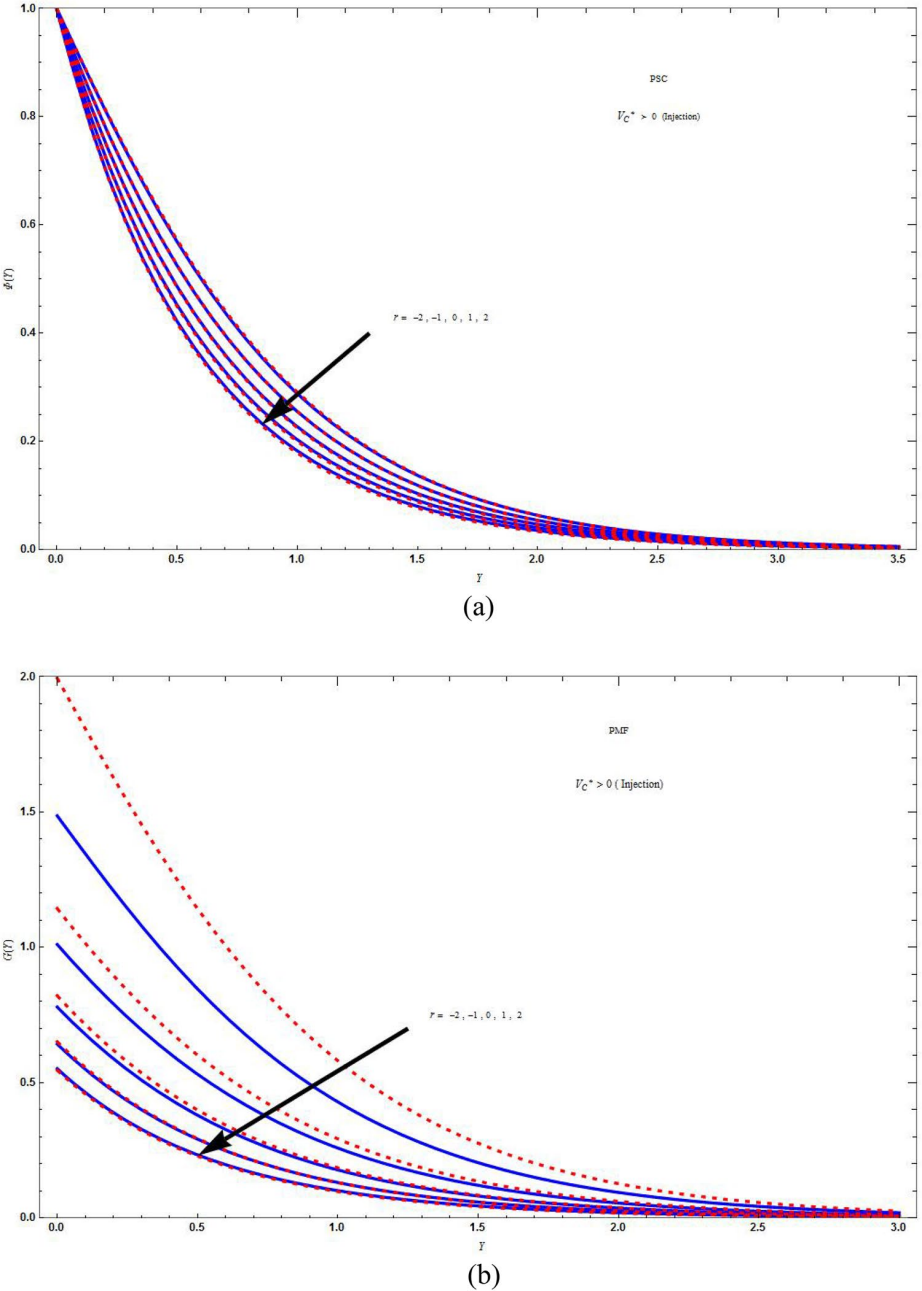
The concentration distribution plot for injection $\left( {V_{C}^{*} = 1} \right)$ boundary with different values of *r* keeping the parameters as $M = C = \Lambda = Sc = K = 1\,,\,J = - 3\,,\,\Delta^{*} = 0.1$ in (**a**) PSC case and in (**b**) PMF case. Blue solid lines are for the base fluid and red dotted lines denotes the hybrid nanofluid $Cu - Al_{2} O_{3} /{\text{water}}$.

Figure 4a and b displays the plot of $\Phi \left( Y \right)$ and *G*(*Y*) verses *Y* for various values of *r* in injection case $V_{C}^{*} > 0$ that is, for mass distribution in PSC and PMF case respectively for various values of mass flux parameter. The consequences of *r* on the concentration in PSC and PMF case are similar. The concentration distribution will be more as the value of *r* raises. The concentration distribution does not vary for base fluid and hybrid nanofluid in PSC case and slight difference in PMF case. As $Y \to 0$ the concentration distribution will be same for any value of *r* and equal to 1 in PSC case and is different for different values of *r* in PMF case then it will become zero at some point of *Y* in both cases.

**Figure 5:**
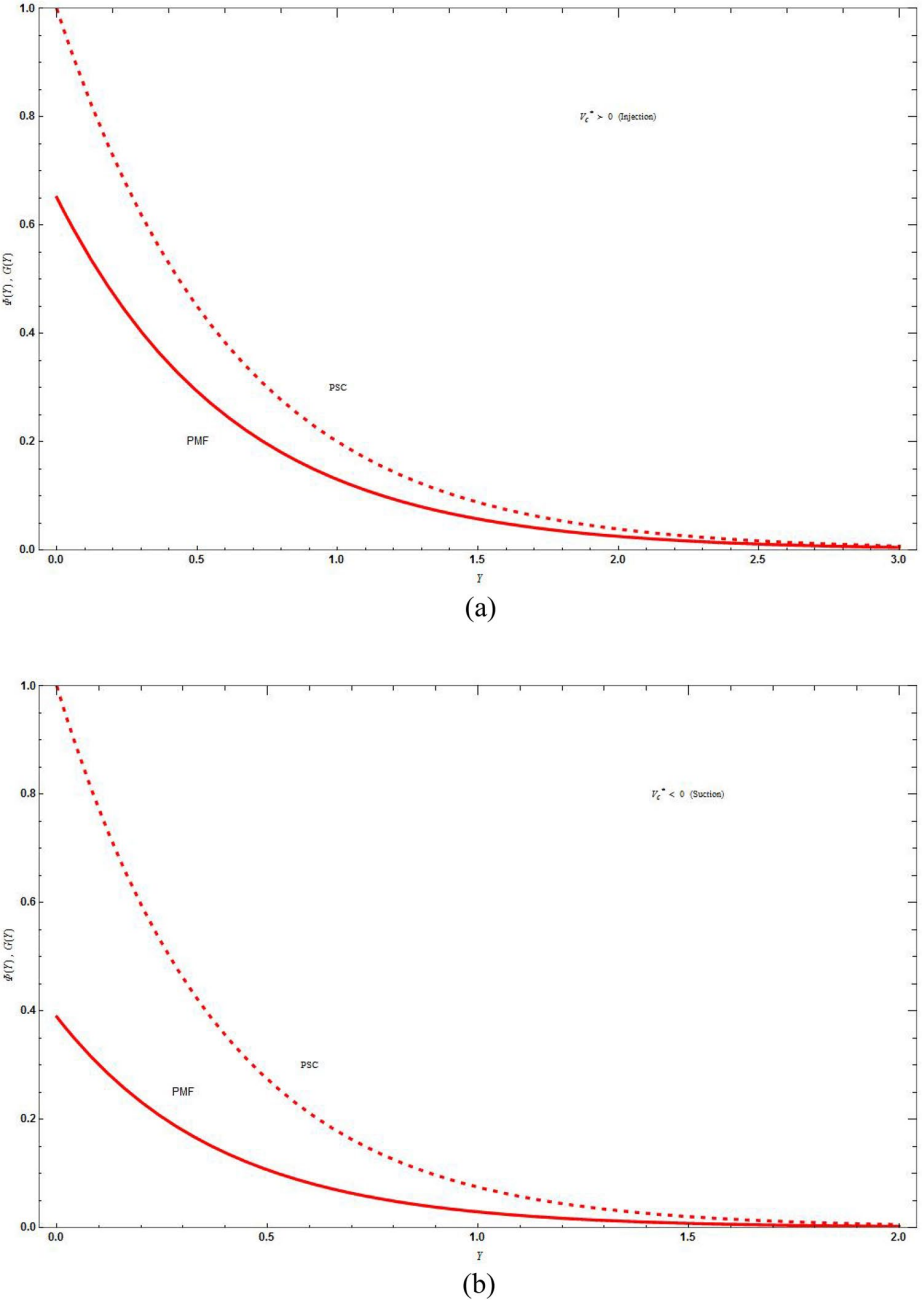
The concentration distribution plot shows the difference between curves of PSC and PMF keeping the parameters as $M = C = \Lambda = Sc = K = r = 1\,,\,\vartheta = - 3\,,\,\Delta^{*} = 0.1$ in (**a**) for injection $\left( {V_{C}^{*} = 1} \right)$ boundary and in (**b**) for suction $\left( {V_{C}^{*} = - 1} \right)$ boundary. Blue solid lines are for the base fluid and red dotted lines denotes the hybrid nanofluid $Cu - Al_{2} O_{3} /{\text{water}}$.

**Figure 6:**
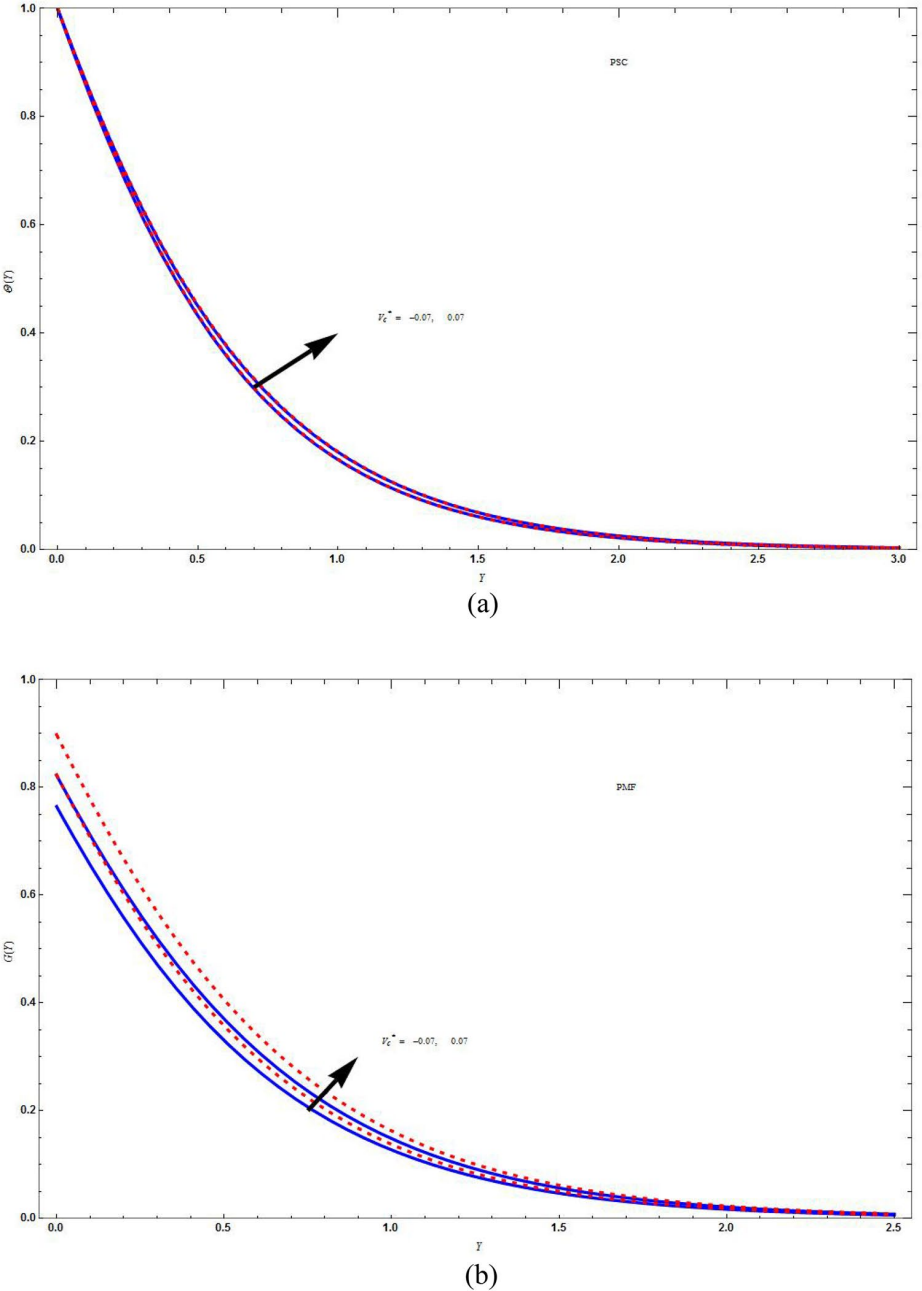
The concentration distribution plot for different $V_{C}^{*}$ keeping the parameters as $M = C = \Lambda = Sc = K = 1\,,\,\vartheta = - 3\,,\,r = - 2\,,\,\Delta^{*} = 0.1$ in (**a**) PSC case and in (**b**) PMF case. Blue solid lines are for the base fluid and red dotted lines denotes the hybrid nanofluid $Cu - Al_{2} O_{3} /{\text{water}}$.

**Figure 7:**
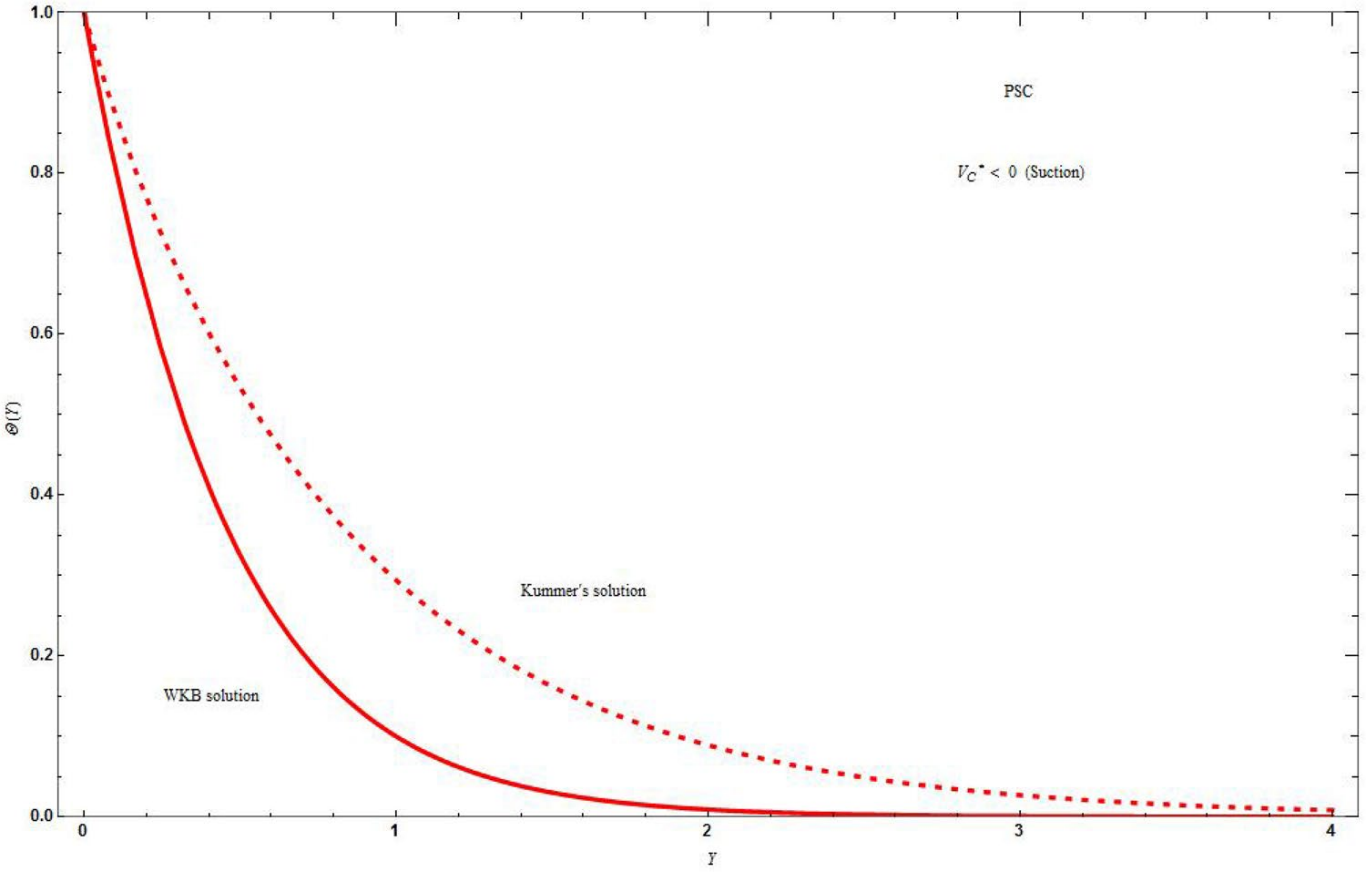
The concentration distribution plot shows the difference between curves of Kummer’s solution and WKB solution in PSC case and suction $\left( {V_{C}^{*} = - 1} \right)$ boundary keeping the parameters as $M = C = \Lambda = Sc = K = r = 1\,,\,\vartheta = - 3\,,\,\Delta^{*} = 0.1$. Blue solid lines are for the base fluid and red dotted lines denotes the hybrid nanofluid $Cu - Al_{2} O_{3} /{\text{water}}$.

**Figure 8:**
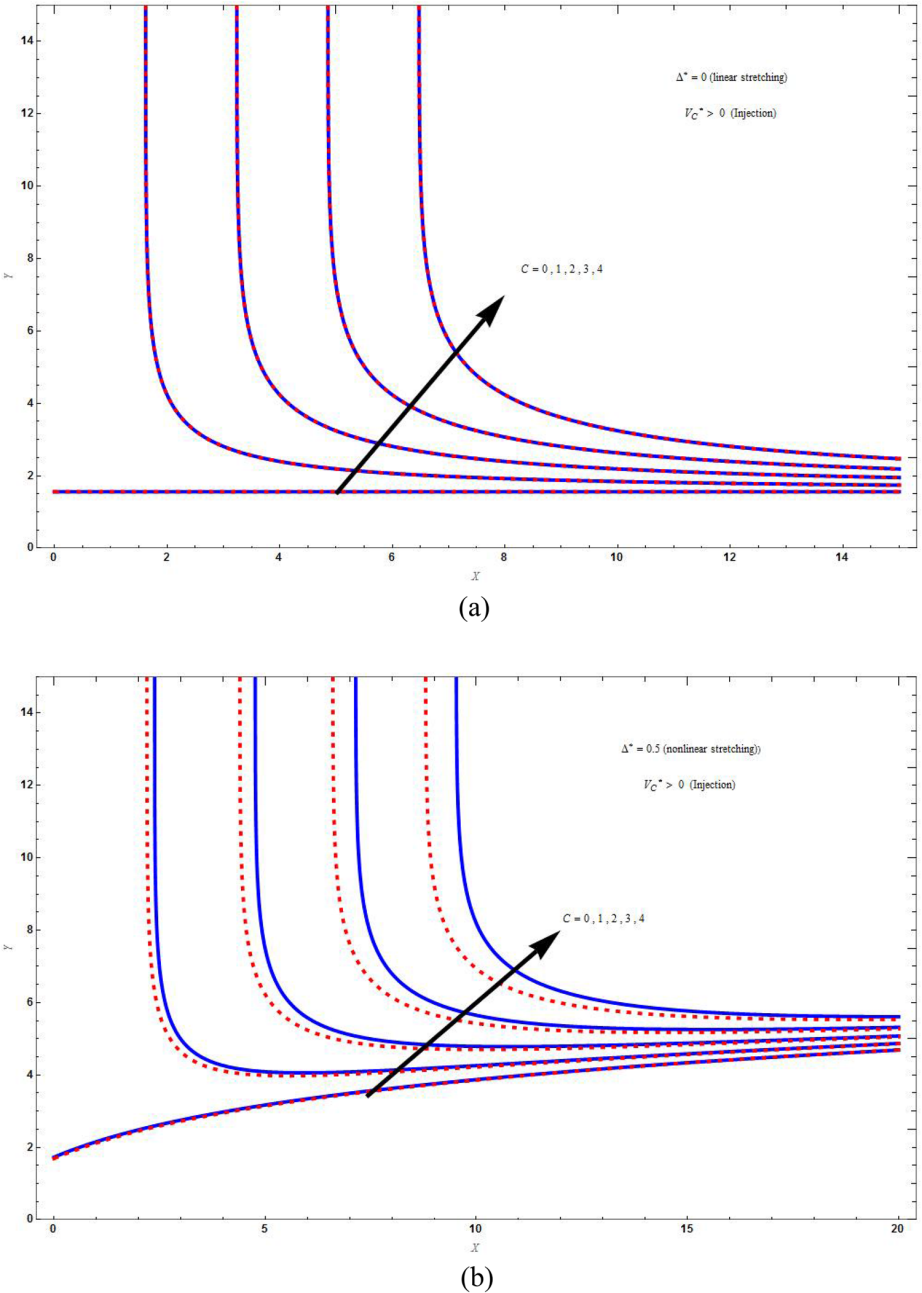
The streamline plot $\psi \left( {X,Y} \right) = C$ for injection boundary $\left( {V_{C}^{*} = 1} \right)$ by varying the constant *C* (**a**) for linear stretching $\left( {\Delta^{*} = 0} \right)$ with $M = K = 0\,,\Lambda = 1\,$, and (**b**) for nonlinear stretching $\left( {\Delta^{*} = 0.5} \right)$ with $M = K = 0.1\,,\Lambda = 1\,$. Blue solid lines are for the base fluid and red dotted lines denotes the hybrid nanofluid $Cu - Al_{2} O_{3} /{\text{water}}$.

Figure 5a and b demonstrate the difference of the concentration distribution in PSC and PMF cases for injection and suction respectively, which shows that the concentration distribution is high for PSC case than for PMF case for both suction and injection. In Fig. 6a and b there is a difference of concentration distribution between suction and injection cases for PSC and PMF respectively. The suction temperature is less compared to the injection temperature both in PSC and PMF cases. In Fig. 7 there is examination of the difference between the temperature distribution of Kummer’s function and WKB asymptotic solution for large Schmidt number in case of PSC for suction velocity. The asymptotic solution cools more compared to the non asymptotic solution, i.e., rate of heat transfer is more for asymptotic solution. Figure 8 depicts the stream line $\psi \left( {X,Y} \right) = C$ for injection case by varying values of *C*. Figure 8a is drawn for linear stretching and Fig. 8b drawn for nonlinear stretching boundary. We can clearly seen that the increasing value of C, will blow up the streamlines, and this will be more for base fluid than for HNF. This is due to the input of external mass flux. The flow is studied with the help of streamline patterns and also the axial and transverse velocity distributions.

## Conclusion

The analysis of the present work is done by finding the exact analytical solution for velocity and non asymptotic solution for temperature distribution in PSC and PMF cases. Further find the asymptotic solution for large Schmidt number by WKB approximation for PSC and PMF cases. Regarding the present work we can give the conclusion as follows,

- Increasing in the rate of super-linear stretching results in enhancement of the liquid lift along downstream
- Streamlines lifted up because of taking nonlinear stretching and are converging at the long distance along downstream
- The liquid lift for base fluid is higher than that of hybrid nanofluid.
- The concentration distribution is high for PSC case than for PMF case in both suction and injection cases.
- The suction temperature is less compared to the injection temperature both in PSC and PMF cases.
- The temperature distribution of asymptotic solution is less compared to the non asymptotic solution.

In the future, we plan to do a similar investigation on a non-Newtonian fluid with heat transfer problems as well. Besides, we feel that adding the effect of velocity mass transpiration and various physical parameters can uncover another interesting phenomenon.

## List of symbols

*a*: Constant, –
*b*: Constant, –
${B}_{0}$: Applied magnetic field, wm^−^^2^
*C*: Concentration, mol m^−3^
$C_{\infty }$: Ambient concentration, mol m^−3^
*D*_*i*_ (*i* = 1 to 3): Effective property ratio, –
*Da*^−1^: Inverse Darcy number, –
*D*_*B*_: Molecular diffusivity, m^2^ s^−1^
*f*: Dimensionless stream function, –
*G*: Dimensionless concentration field in PMF case mol m^−3^
*K*: Porous permeability, Hm^−1^
*k*_*C*_: Chemical reaction parameter, –
*M*: Magnetic field parameter, w S kg^−1^
Sc: Schmidt number, –
*u* and *v*: Velocity components, ms^−1^
${\text{V}}_{C}^{*} < 0/ > 0$: Suction/injection parameter, –
*x, y*: Cartesian coordinates, m

## Greek symbols

$$\eta$$: Similarity variable, –
$$\psi$$: Stream function, –
*μ*: Dynamic viscosity, kg m^−1^ s^−1^
*ν*: Kinematic viscosity, m^2^ s^−1^
*ρ*: Density, kg m^−3^
$\vartheta$: Chemical reaction parameter, –
*σ*: Electrical conductivity, Sm^−1^
$\sigma^{*}$: Stefan–Boltzmann constant, Wm^−2^ K^−4^
$$\Lambda$$: Brinkman ratio, –
$\phi$: Dimensionless concentration, mol m^−3^
$\varphi_{1} \,\,\& \,\,\varphi_{2}$: Solid particle volume fraction of the aluminum oxide and copper, respectively, –
$\Phi$: Dimensionless concentration field in PSC case mol m^−3^

## Subscript

*hnf*: Hybrid nanofluid
*s*: Solid particle involved in hybrid nanofluid
*f*: Base fluid
*eff*: Effective

## Abbreviations

B.Cs: Boundary conditions
HNF: Hybrid nanofluid
MHD: Magnetohydrodynamic
Al_2_O_3_: Aluminum oxide (alumina)
Cu: Copper
H_2_O: Pure water
